# Correlation Between Endothelial Morphology, Ocular Biometric Parameters, and Systemic Comorbidities in a Large Caucasian Cohort

**DOI:** 10.3390/jcm15155815

**Published:** 2026-07-25

**Authors:** Maria Martinez-de-la-Casa, Maria Matilla, Javier Garcia-Bella, Laura Morales Fernandez, Julian Garcia-Feijoo, Jose M. Martínez-de-la-Casa, Barbara Burgos-Blasco

**Affiliations:** 1 Department of Ophthalmology, Hospital Clínico San Carlos, Universidad Complutense de Madrid, Calle del Profesor Martín Lagos s/n, 28040 Madrid, Spain; mariamartinezcasa@gmail.com (M.M.-d.-l.-C.); javier.garciabll@gmail.com (J.G.-B.); lauramoralesfernandez@gmail.com (L.M.F.); jgarciafeijoo@hotmail.com (J.G.-F.);; 2 Department of Ophthalmology, Hospital Universitario Fundación Alcorcón, Calle Budapest 1, 28922 Alcorcón, Spain; mariamatillar@gmail.com

**Keywords:** corneal endothelium, endothelial cell density, ocular biometry, axial length, aging, specular microscopy

## Abstract

**Background/Objectives:** To evaluate the association between demographic, ocular biometric, and systemic comorbidity variables with corneal endothelial morphometric parameters in a Caucasian adult population undergoing cataract surgery. **Methods:** Cross-sectional study in a large cohort of patients who were candidates for cataract surgery with no other concomitant ocular pathology. Endothelial cell density (ECD), hexagonality (HEX), the coefficient of variation (CV), and the presence of guttae were assessed using specular microscopy (Tomey EM-4000). Biometric parameters were obtained by partial coherence interferometry (IOLMaster 700), and systemic comorbidities were recorded. Multiple linear regression models adjusted for age and sex were applied, along with stratified analyses according to axial length (AL). **Results:** A total of 1032 eyes from 1032 patients were included. The mean age was 75.9 ± 9.3 years. The cohort comprised 366 men (35.5%) and 666 women (64.5%). The mean ECD was 2113 ± 544 cells/mm^2^, HEX 42.7 ± 21.6%, and CV 43.2 ± 9.6%. The prevalence of guttae was 15.4% (159 eyes). ECD correlated negatively with age (r = −0.148; *p* < 0.001) and positively with central corneal thickness (r = 0.091; *p* = 0.004) and anterior chamber depth (r = 0.064; *p* = 0.030). In the multivariate model, age was independently associated with lower ECD (β = −8.40 cells/mm^2^/year; *p* < 0.001), whereas male sex was associated with higher ECD and lower CV. No significant associations were found with AL, keratometry, or systemic comorbidities. Stratified analysis by AL group showed consistent patterns with no relevant differences. **Conclusions:** Corneal endothelial morphometry in Caucasian adults is primarily associated with age, sex, central corneal thickness, and anterior chamber depth, with no significant association with axial length or systemic comorbidities.

## 1. Introduction

The corneal endothelium is a monolayer of non-regenerative polygonal cells whose primary role is to maintain corneal transparency through a delicate osmotic balance between the stroma and the aqueous humor [1,2]. Its structural and functional integrity is essential for preserving visual acuity and is particularly threatened under surgical, inflammatory, or metabolic stress [3]. Unlike other corneal layers, the adult human endothelium lacks meaningful proliferative capacity. Therefore, the progressive cell loss over time is compensated by cellular hypertrophy (polymegatism) and changes in cell shape (pleomorphism), which can compromise long-term function [4,5,6].

Numerous factors have been proposed as modulators of endothelial morphology and endothelial cell density (ECD), including age, sex, anterior segment biometric dimensions, and a variety of systemic conditions. A physiological decline in ECD with aging has been described, together with an increase in the coefficient of variation (CV) and a reduction in hexagonality (HEX), the latter two being indicators of progressive endothelial dysfunction [7,8,9]. The relationship between certain comorbidities such as diabetes mellitus (DM) [10,11], arterial hypertension, or thyroid dysfunction and endothelial structural deterioration has also been explored, although many reports are limited by sample size, population heterogeneity, or inconsistent results [12].

In addition, ocular biometric characteristics such as central corneal thickness (CCT), anterior chamber depth (ACD), lens thickness (LT), and, most notably, axial length (AL) have been implicated as anatomical factors potentially associated with endothelial status [13]. Several Asian and Latin American studies have investigated these associations, but data from Caucasian populations remain scarce. Extrapolating findings from other ethnicities to Caucasians may be inappropriate given known differences in ocular morphology and endothelial behavior across groups.

Within this context, the available scientific literature on determinants of corneal endothelial morphology in Caucasian populations is still limited. Moreover, most published studies include small samples, focus on specific diseases, or consider only a reduced set of clinical variables. Very few investigations have simultaneously evaluated the effects of clinical, biometric, and systemic variables on the three classic parameters of endothelial morphology (ECD, HEX, and CV) using high-precision instrumentation such as partial coherence interferometry and automated specular microscopy.

The primary objective of this study was to assess the association of demographic, biometric, and systemic comorbidity variables with corneal endothelial morphometric parameters in a large cohort of Caucasian patients scheduled for cataract surgery. To the best of our knowledge, this is one of the largest studies of its kind conducted in a Caucasian population.

## 2. Materials and Methods

### 2.1. Study Design and Population

We conducted a cross-sectional study of consecutive patients assessed at the cataract surgery clinic of the Ophthalmology Department, Hospital Clínico San Carlos (Madrid, Spain) between January 2022 and December 2023. The study was approved by the Research Ethics Committee for Medicinal Products (CEIm) of Hospital Clínico San Carlos, Madrid, Spain (reference number CEIm-HCSC 25/137) and adhered to the principles of the Declaration of Helsinki. Given the nature of the study, the Ethics Committee waived the requirement for individual informed consent; data confidentiality was guaranteed throughout. This observational study was reported in accordance with the STROBE (Strengthening the Reporting of Observational Studies in Epidemiology) guidelines.

We included eyes of consecutive Caucasian patients ≥18 years old with a surgical indication for cataract and complete biometric and endothelial data. Exclusion criteria were any previous intraocular or corneal surgery (including refractive and cataract surgery); corneal pathology such as bullous keratopathy, keratoconus or other corneal ectasias, corneal opacities or scarring, and previous corneal trauma; any form of glaucoma or treated ocular hypertension; a history of uveitis or intraocular inflammation; and poor-quality specular microscopy images that precluded reliable automated cell analysis. When both eyes met the criteria, the right eye was selected before any statistical analysis, in order to preserve the statistical independence of the observations. Ethnicity was ascertained from the self-reported information recorded in the electronic medical record and confirmed during the pre-surgical interview. The analysis was restricted to Caucasian patients because endothelial cell density and morphometry are known to differ across ethnic groups and because most published normative data derive from Asian and Latin American cohorts; a homogeneous Caucasian sample therefore minimizes ethnicity-related confounding and helps address the scarcity of reference data in this population.

### 2.2. Variables Recorded

Age, sex, and relevant comorbidities were recorded: hypertension (HBP), DM, dyslipidemia (DL), anticoagulant/antiplatelet therapy, thyroid disease, and benign prostatic hyperplasia (BPH) in men.

Ocular biometry was obtained by partial coherence interferometry (IOLMaster 700, Carl Zeiss Meditec AG, Jena, Germany) and included AL, LT, ACD, white-to-white (WTW), aqueous depth (AQD), anterior and posterior corneal keratometry (flat K1 and steep K2 on both surfaces), and CCT. From K1 and K2, mean anterior and posterior curvatures were calculated as (anterior K1 + anterior K2)/2 and (posterior K1 + posterior K2)/2, respectively. Total mean keratometry (Km) was then computed as the average of the anterior and posterior means: (anterior Km + posterior Km)/2.

Endothelial morphology was analyzed with non-contact specular microscopy (Tomey EM-4000, Tomey GmbH, Nuremberg, Germany), obtaining ECD (cells/mm^2^), HEX (%), CV (%), and the presence or absence of guttae.

### 2.3. Axial Length Classification

To explore the impact of globe size, eyes were categorized by AL into three groups: short eyes (AL < 22 mm), medium eyes (AL ≥ 22 mm and <24 mm), and long eyes (AL ≥ 24 mm).

### 2.4. Statistical Analysis

Analyses were performed with SPSS version 29.0.2 (IBM Corp., Armonk, NY, USA). Continuous variables with normal distribution are reported as mean ± standard deviation (SD); non-normal variables are reported as median and interquartile range. Normality was assessed with the Kolmogorov–Smirnov test. Parametric tests (Student’s t, ANOVA with pairwise comparisons when appropriate) were used for normally distributed variables, and non-parametric tests (Mann–Whitney U, Kruskal–Wallis) for non-normal variables. Separate multiple linear regression models were fitted for each endothelial parameter, considering clinical and biometric variables as predictors. Analyses were then repeated after stratification by AL group. A *p*-value < 0.05 was considered statistically significant.

## 3. Results

We included 1032 eyes from 1032 patients. The mean age was 75.9 ± 9.3 years. The cohort comprised 366 men (35.5%) and 666 women (64.5%). Regarding systemic comorbidities, 223 patients (21.6%) had DM, 401 (38.9%) HBP, and 172 (16.7%) DL; 101 (9.8%) were on anticoagulant or antiplatelet therapy. Thyroid dysfunction was present in 69 patients (6.7%) and BPH in 87 men (8.4%) (Table 1).

### 3.1. Ocular Biometric Parameters

The mean AL was 23.6 ± 1.5 mm (range, 19.8–34.6 mm). ACD was 3.1 ± 0.4 mm, AQD was 2.5 ± 0.4 mm, and LT was 4.7 ± 0.4 mm. CCT measured 543.9 ± 40.1 µm, and the white-to-white (WTW) diameter was 11.8 ± 0.4 mm.

For corneal curvature, the anterior surface showed a mean K1 = 43.6 ± 1.6 D and K2 = 44.6 ± 1.6 D, with anterior astigmatism of 0.9 ± 0.8 D. The posterior surface showed an absolute mean curvature of K1 = 48.6 ± 1.9 D and K2 = 50.4 ± 2.1 D, with posterior astigmatism of 1.8 ± 1.1 D. Total curvature was K1 = 43.6 ± 1.6 D and K2 = 44.7 ± 1.6 D, with total astigmatism of 1.0 ± 0.8 D (Table 2).

### 3.2. Endothelial Characteristics

Overall endothelial parameters were: mean ECD 2113 ± 544 cells/mm^2^, mean HEX 42.7 ± 21.6%, and CV 43.2 ± 9.6%. The prevalence of guttae was 15.4% (159 eyes) (Table 3).

### 3.3. Bivariate Correlations

**Figure 1:**
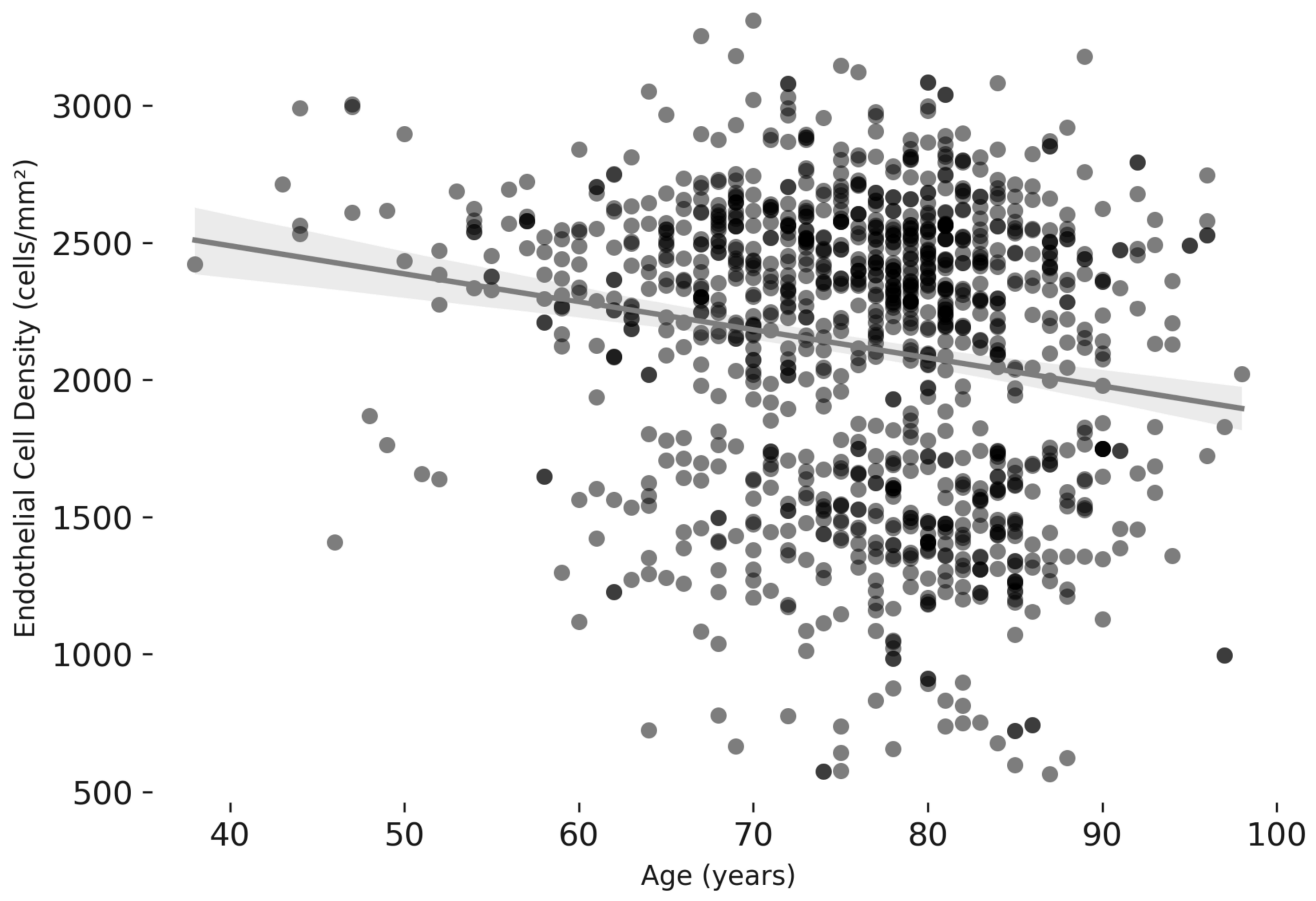
Correlation between age and endothelial cell density. Scatterplot showing the relationship between patient age and corneal endothelial cell density. A significant negative correlation was observed, indicating a gradual decrease in endothelial cell density with increasing age.

**Figure 2:**
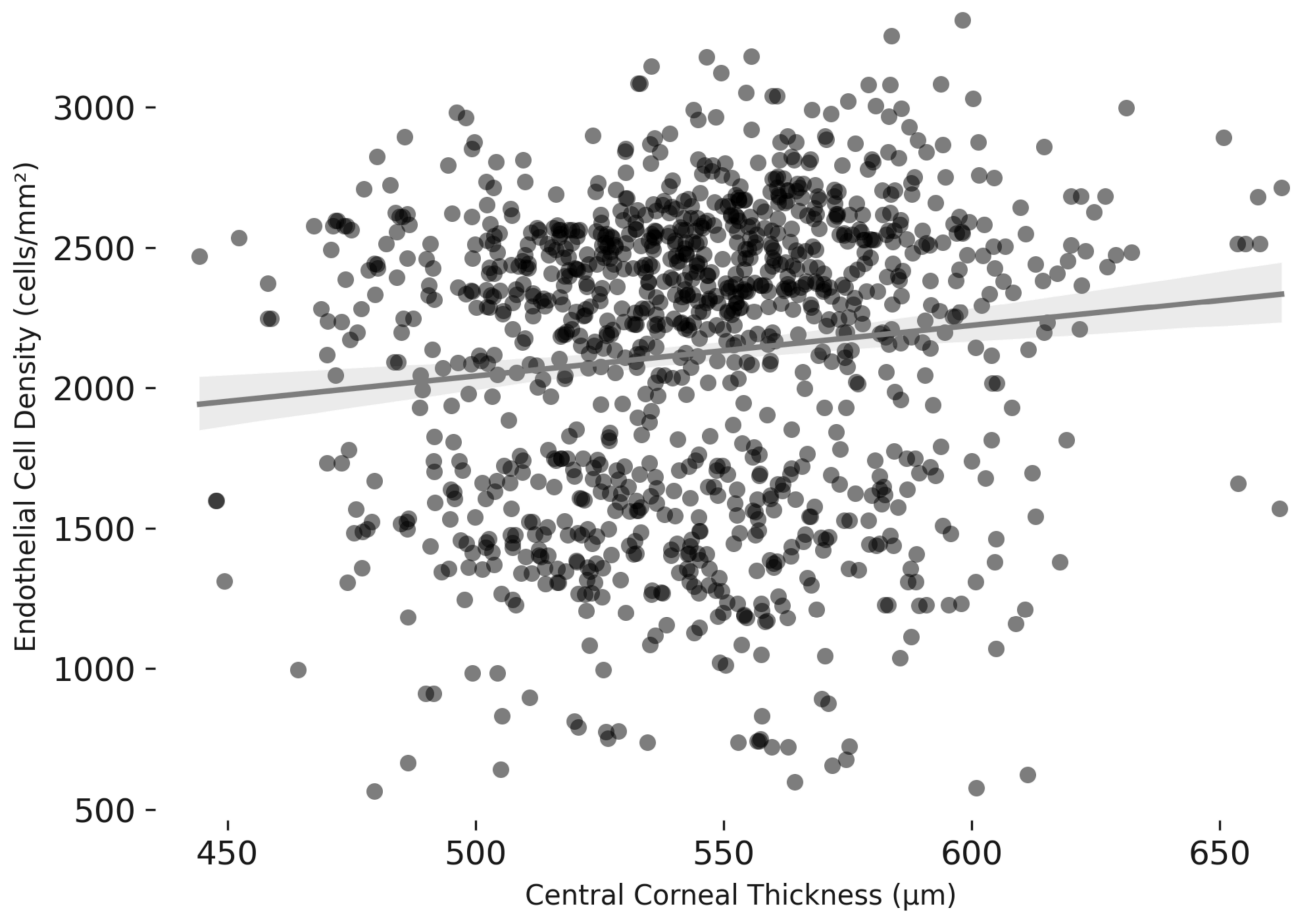
Correlation between central corneal thickness and endothelial cell density. Scatterplot illustrating the relationship between central corneal thickness (CCT) and endothelial cell density.

**Figure 3:**
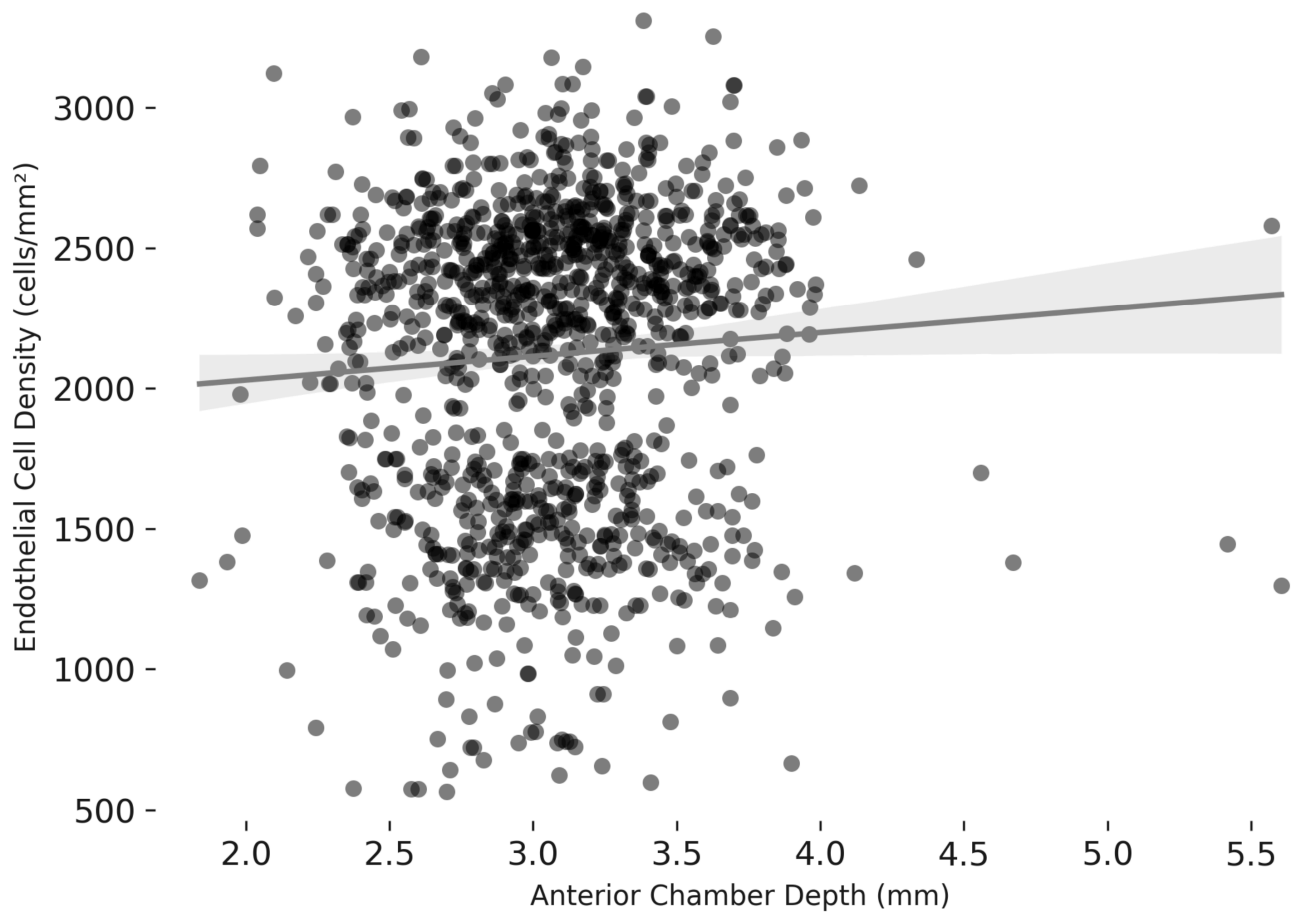
Correlation between anterior chamber depth and endothelial cell density. Scatterplot showing the relationship between anterior chamber depth (ACD) and corneal endothelial cell density.

ECD showed a significant negative correlation with age (r = −0.148, *p* < 0.001) (Figure 1), equivalent to an approximate loss of 84 cells/mm^2^ per decade, and positive correlations with CCT (r = 0.091, *p* = 0.004) (Figure 2) and ACD (r = 0.064, *p* = 0.030) (Figure 3). No associations were observed with AL, LT, or keratometric parameters.

CV correlated positively with age (r = 0.091, *p* = 0.003), anterior astigmatism (r = 0.066, *p* = 0.034), and total astigmatism (r = 0.069, *p* = 0.027), and negatively with CCT (r = −0.072, *p* = 0.021). HEX showed no significant correlations with any variable.

By sex, men had higher ECD than women (2161 ± 544 vs. 2087 ± 531 cells/mm^2^; *p* = 0.036) and lower CV (42.1 ± 9.6% vs. 43.8 ± 9.3%; *p* = 0.005). No significant differences were observed for HEX with respect to DM, HBP, DL, anticoagulant therapy, thyroid dysfunction, or BPH (all *p* ≥ 0.05).

### 3.4. Multivariate Models

In multivariate models adjusted for age and sex, ECD was independently associated with age (β = −8.40 cells/mm^2^ per year; 95% CI −12.5 to −4.30; *p* < 0.001), whereas the remaining covariates (including AL, keratometry, CCT, WTW, and comorbidities) did not reach statistical significance. For HEX, only LT showed a weak positive association (β = 5.07% per mm; 95% CI 0.05 to 10.1; *p* = 0.048), with limited overall fit. For CV, there was a positive association with age (β = 0.12; 95% CI 0.05 to 0.20; *p* = 0.001) and with female sex (β = 1.71; 95% CI 0.37 to 3.06; *p* = 0.013), along with a negative association with Km (β = −0.70; 95% CI −1.22 to −0.18; *p* = 0.009).

In the logistic regression model for the presence of guttae (n = 1004), adjusted for age, sex, and comorbidities, no predictor reached statistical significance (all *p* ≥ 0.17). The overall fit was low (McFadden’s pseudo-R^2^ = 0.006).

### 3.5. Stratified Analysis by Axial Length

The cohort was divided by AL into short (<22 mm; n = 54), medium (≥22 and <24 mm; n = 702), and long (≥24 mm; n = 276) groups (Table 4).

In the medium-AL group, which comprised 68.0% of the sample, the overall cohort pattern was maintained. ECD correlated negatively with age (r = −0.17; *p* < 0.001) and showed a positive trend with CCT (r = 0.07; *p* = 0.07). CV was positively associated with age (r = 0.09; *p* = 0.021).

In long eyes (≥24 mm), the ECD–age relationship was not significant (r = −0.06; *p* = 0.34). ECD correlated positively with WTW (r = 0.155; *p* = 0.009). CV correlated with age (r = 0.114; *p* = 0.055) and showed associations with total curvature (K1: r = −0.194; *p* = 0.001; K2: r = −0.110; *p* = 0.065) and with total astigmatism (r = 0.147; *p* = 0.013).

In short eyes (<22 mm), ECD showed a negative trend with age (r = −0.172; *p* = 0.22). There was a positive trend between ECD and ACD (r = 0.07; *p* = 0.063) and between ECD and AQD (r = 0.05; *p* = 0.69). CV correlated negatively with WTW (r = −0.18; *p* = 0.21).

## 4. Discussion

This study provides a detailed analysis of corneal biometric and endothelial parameters in a large cohort of Caucasian adults. The findings confirm the association of age, sex, central corneal thickness, and ACD with endothelial morphometry, as well as the absence of a significant association with AL or keratometry. The progressive loss of ECD with aging and the greater cell variability in women are consistent with the physiological aging pattern described in Asian and European populations [14,15,16,17]. These results reinforce the clinical utility of endothelial measurements as indicators of corneal integrity. As our cohort was exclusively Caucasian, these data contribute Caucasian-specific reference values and caution against extrapolating normative thresholds obtained from Asian or Latin American populations; they do not, however, provide normative values for other ethnic groups.

In line with studies conducted in Chinese and Malay populations, ECD showed an inverse relationship with age, reflecting gradual impairment of the endothelial pump and compensatory enlargement and polymegatism of the remaining cells. Wang et al. reported an average reduction of 36 cells/mm^2^ per year, mainly associated with CCT [5], whereas Norhani et al. observed a similar decrease for each millimeter of axial elongation [6]. Our results confirm a negative correlation between ECD and age, but not with AL, suggesting that in the adult population age-related degeneration predominates over ocular morphometry.

It should be emphasized that, although several of these associations reached statistical significance, the corresponding correlation coefficients were weak (|r| < 0.15 for age, central corneal thickness, and anterior chamber depth). Their significance is largely driven by the large sample size, and their clinical relevance is limited. The estimated loss of approximately 84 cells/mm^2^ per decade corresponds to only about 4% of the mean endothelial cell density across an adult lifetime, a magnitude unlikely to be clinically meaningful in eyes without additional endothelial stressors. These associations should therefore be regarded as descriptive population-level trends rather than clinically actionable predictors at the individual level.

By contrast, in younger populations with axial myopia, a significant relationship has been described between globe elongation and reduced ECD. Tananuvat and Khumchoo reported that highly myopic eyes had lower density and higher CV, findings attributed to cellular redistribution secondary to corneal stretching [8]. The absence of this association in our series may reflect structural stabilization of the anterior segment in adulthood and the predominance of age-related over morphometric factors in determining endothelial parameters.

Regarding corneal thickness, the positive correlation between ECD and CCT agrees with reports from Chinese and Thai populations. This link is explained by the function of the endothelial Na^+^/K^+^-ATPase pump, whose impairment is associated with stromal edema and a compensatory increase in CCT. However, the normal thickness range in our cohort suggests that the observed endothelial changes reflect a physiological rather than a pathological process.

In the present study we observed a weak but statistically significant positive correlation between ACD and ECD. This finding suggests that eyes with slightly deeper anterior chambers tend to have higher ECD, although the magnitude of the effect is clinically modest. The association may be explained, at least in part, by anatomical and biomechanical factors: a wider anterior chamber could offer greater protection against mechanical or thermal stress on the endothelium, reducing the risk of cellular damage. However, after adjustment for age and axial length, the relationship lost significance, indicating that age remains the main determinant of endothelial loss and that the influence of ACD is likely secondary or dependent on other ocular factors.

Furthermore, the lack of a significant association between HEX and biometric variables is consistent with the report by Pekel et al., who demonstrated that the percentage of hexagonal cells correlates mainly with ACD and volume, but not with corneal curvature or age [9]. These results support the idea that HEX reflects the morphological stability of the endothelium rather than anatomical variations in the eye.

In the systemic context, several studies have evaluated the effect of type 2 DM on the corneal endothelium [18,19], showing a tendency toward lower density and HEX and higher CV, although statistical significance is not always achieved. Chowdhury et al. noted that poor glycemic control and the presence of diabetic nephropathy were associated with greater polymegatism [12]. In our study, we did not detect significant differences in endothelial parameters between patients with and without the systemic comorbidities examined. However, this should not be interpreted as evidence that these conditions have no effect on the endothelium. Comorbidities were recorded only as present or absent, without data on disease duration, severity, or degree of control, and only a limited number of common conditions were assessed, whereas less prevalent disorders such as chronic kidney disease or autoimmune conditions were not evaluated. The study may also have been underpowered to detect small effects, particularly within subgroups. A limited or undetectable effect is therefore compatible with well-controlled or recently diagnosed disease but does not exclude clinically relevant effects in poorly controlled or long-standing conditions.

The analysis stratified by AL revealed a differential pattern among groups. In long eyes, a negative correlation was observed between CV and total corneal curvature, consistent with the corneal flattening described in myopic eyes. In short and medium eyes, associations were weak or non-significant, in line with the structural heterogeneity of the endothelium as a function of globe size. However, the short-eye subgroup was small (n = 54) and considerably smaller than the medium-eye group (n = 702); the non-significant endothelial cell density–age correlation observed in short eyes therefore most likely reflects insufficient statistical power rather than a genuine absence of association, and should be interpreted with caution. The concordance of these findings with studies performed in different ethnicities suggests that anatomical variations related to AL do not substantially modify endothelial morphology in the absence of ocular pathology [13,20,21].

Among possible pathophysiological explanations, the progressive loss of endothelial cells may result from a combination of apoptosis induced by oxidative stress, reduced regenerative capacity, and cumulative microtrauma associated with aging [22,23]. The higher density observed in men could be related to hormonal differences or differential exposure to environmental factors, whereas the increase in CV in women may reflect a more marked cellular remodeling process, in keeping with previous observations in Asian cohorts [24,25,26].

This study has several strengths, including its large sample size, single-center design with uniform methodological control, and the availability of complete biometric variables, which enabled a robust multivariate analysis. To the best of our knowledge, this is one of the largest studies of its kind conducted in a Caucasian population.

### Limitations

Several limitations should be acknowledged. First, the cross-sectional design precludes any causal inference; the reported associations describe co-variation at a single point in time and cannot establish that age or any other factor causes endothelial change. Second, and importantly, the study population consisted exclusively of patients scheduled for cataract surgery. Because cataract is strongly age-dependent, older individuals are over-represented, the age range is relatively narrow, and lens opacity itself may influence some optical measurements; consequently, the findings may not be generalizable to younger individuals or to eyes without cataract, and this should be regarded as a major limitation. Third, the axial-length subgroups were markedly unbalanced, with only 54 short eyes, which limited the statistical power of the subgroup analyses. Fourth, systemic comorbidities were recorded dichotomously, without information on duration, severity, or degree of control, and several potentially relevant conditions (for example chronic kidney disease or autoimmune disorders) were not assessed. Fifth, the single-center design may limit generalizability. Finally, the observed correlations, although statistically significant, were weak and of limited clinical magnitude. Future multicenter and longitudinal studies that also include non-cataract and younger populations and incorporate graded comorbidity data could assess the temporal dynamics of endothelial cell loss and clarify potential differences between Caucasian and other populations.

## 5. Conclusions

In this large Caucasian cohort, corneal endothelial morphometry was primarily associated with age, sex, central corneal thickness, and anterior chamber depth, whereas axial length, keratometry, and the systemic comorbidities examined were not significantly associated with endothelial parameters. The absence of a detectable association with comorbidities should be interpreted cautiously, as it may reflect limited statistical power or the lack of severity data rather than a true lack of effect. These findings support the use of age-adjusted, population-specific endothelial reference values in the clinical assessment of patients prior to cataract surgery and provide a basis for future longitudinal research in Caucasian populations.

## Figures and Tables

**Table 1:** Demographic and systemic characteristics of the study population. Descriptive statistics of the study sample, including age, sex distribution, and prevalence of systemic comorbidities. Values are expressed as mean ± standard deviation (SD) for continuous variables and absolute numbers (percentages) for categorical variables.

Parameter	Value
Age (years), mean ± SD	75.9 ± 9.3
**Sex**	
Male	366 (35.5%)
Female	666 (64.5%)
**Systemic comorbidities**	
Diabetes mellitus	223 (21.6%)
Hypertension	401 (38.9%)
Dyslipidemia	172 (16.7%)
Anticoagulant/antiplatelet therapy	101 (9.8%)
Thyroid disease	69 (6.7%)
Benign prostatic hyperplasia	87 (8.4%)

**Table 2:** Biometric parameters by sex. Comparison of anterior segment biometric parameters between male and female eyes. Data are presented as mean ± SD. The *p*-values correspond to Student’s *t*-test or Mann–Whitney U test, depending on variable distribution.

Variable	Male (Mean ± SD)	Female (Mean ± SD)	*p*-Value
Mean anterior Km (D)	43.61 ± 1.55	44.30 ± 1.48	<0.001
Mean posterior Km (D)	48.86 ± 1.86	49.79 ± 1.97	<0.001
Mean total Km (D)	43.68 ± 1.58	44.36 ± 1.50	<0.001
Anterior chamber depth, ACD (mm)	3.18 ± 0.39	3.01 ± 0.41	<0.001
Endothelium–lens distance, AQD (mm)	2.63 ± 0.39	2.47 ± 0.42	<0.001
Lens thickness, LT (mm)	4.64 ± 0.45	4.65 ± 0.40	0.488
Central corneal thickness, CCT (µm)	547.36 ± 34.57	541.80 ± 42.15	0.025
Corneal diameter, WTW (mm)	11.98 ± 0.41	11.77 ± 0.40	<0.001

**Table 3:** Endothelial parameters by sex. Comparison of corneal endothelial morphology between male and female eyes. Data are expressed as mean ± SD. Statistical significance was assessed using Student’s *t*-test or Mann–Whitney U test according to normality.

Variable	Male (Mean ± SD)	Female (Mean ± SD)	*p*-Value
Endothelial cell density, ECD (cells/mm^2^)	2161.3 ± 544.2	2087.18 ± 531.24	0.036
Hexagonality, HEX (%)	42.83 ± 9.65	42.78 ± 24.70	0.08
Coefficient of variation, CV (%)	42.1 ± 9.9	43.8 ± 9.3	0.005

**Table 4:** Endothelial parameters according to axial length group. Comparison of corneal endothelial parameters among short (<22 mm), medium (22–24 mm), and long (≥24 mm) eyes. Values are presented as mean ± standard deviation (SD) and sample size (n). Statistical significance was assessed using the Kruskal–Wallis test.

Variable	Short (<22 mm)	Medium (22–24 mm)	Long (≥24 mm)	*p*-Value
ECD (cells/mm^2^)	1999.9 ± 569.1 (n = 54)	2110.8 ± 532.0 (n = 702)	2157.1 ± 559.8 (n = 276)	0.027
HEX (%)	48.5 ± 51.9 (n = 54)	42.1 ± 9.1 (n = 702)	43.3 ± 28.0 (n = 276)	0.937
CV (%)	44.4 ± 9.5 (n = 54)	43.1 ± 8.8 (n = 702)	43.3 ± 11.2 (n = 276)	0.572

## Data Availability

The data presented in this study are available on request from the corresponding author. The data are not publicly available due to privacy and ethical restrictions.
